# miRNAs and SAMHD1 regulation in vitro and in a model of HIV CNS disease

**DOI:** 10.1186/s12974-015-0380-y

**Published:** 2015-09-04

**Authors:** Kenneth W. Witwer, Erin L. Buchanan, Stephanie L. Myers, Melissa A. McAlexander

**Affiliations:** Department of Molecular and Comparative Pathobiology, The Johns Hopkins University School of Medicine, 733 North Broadway, BRB Suite 831, Baltimore, MD 21025 USA

## Abstract

Pilakka-Kanthikeel et al. recently reported higher levels of the retroviral restriction factor sterile alpha motif and histidine/aspartic acid domain-containing protein 1 (SAMHD1) in astrocytes than in microglia, suggesting that SAMHD1 levels might explain in part the relatively refractory nature of astrocytes to retroviral replication. These findings are consistent with our studies of simian and human immunodeficiency virus infection of astrocytes and macrophages. Similarly, a role for two host microRNAs in post-transcriptional regulation of SAMHD1 agrees with our in vitro results and those of others. However, data from an animal model of HIV neurologic disorders may not be consistent with robust miRNA-mediated regulation of SAMHD1 in vivo.

## Letter to the editor

We were pleased to read the recent article of Pilakka-Kanthikeel and colleagues, reporting higher levels of the retroviral restriction factor sterile alpha motif and histidine/aspartic acid domain-containing protein 1 (SAMHD1) in astrocytes than in microglia [1]. The data suggest that SAMHD1 levels might explain in part the relatively refractory nature of astrocytes to retroviral replication. These findings are consistent with our studies of simian and human immunodeficiency virus (SIV and HIV) infection of astrocytes and macrophages ([2], "Regulation of the SAMHD1 transcript during retroviral infection of the central nervous system," submitted); see also the different results on SAMHD1 in elite suppressor populations [3-5]. Similarly, a role for two host microRNAs (miRNAs) in post-transcriptional regulation of SAMHD1 agrees with our results [2] and Swaminathan et al. [6].

**Fig. 1:**
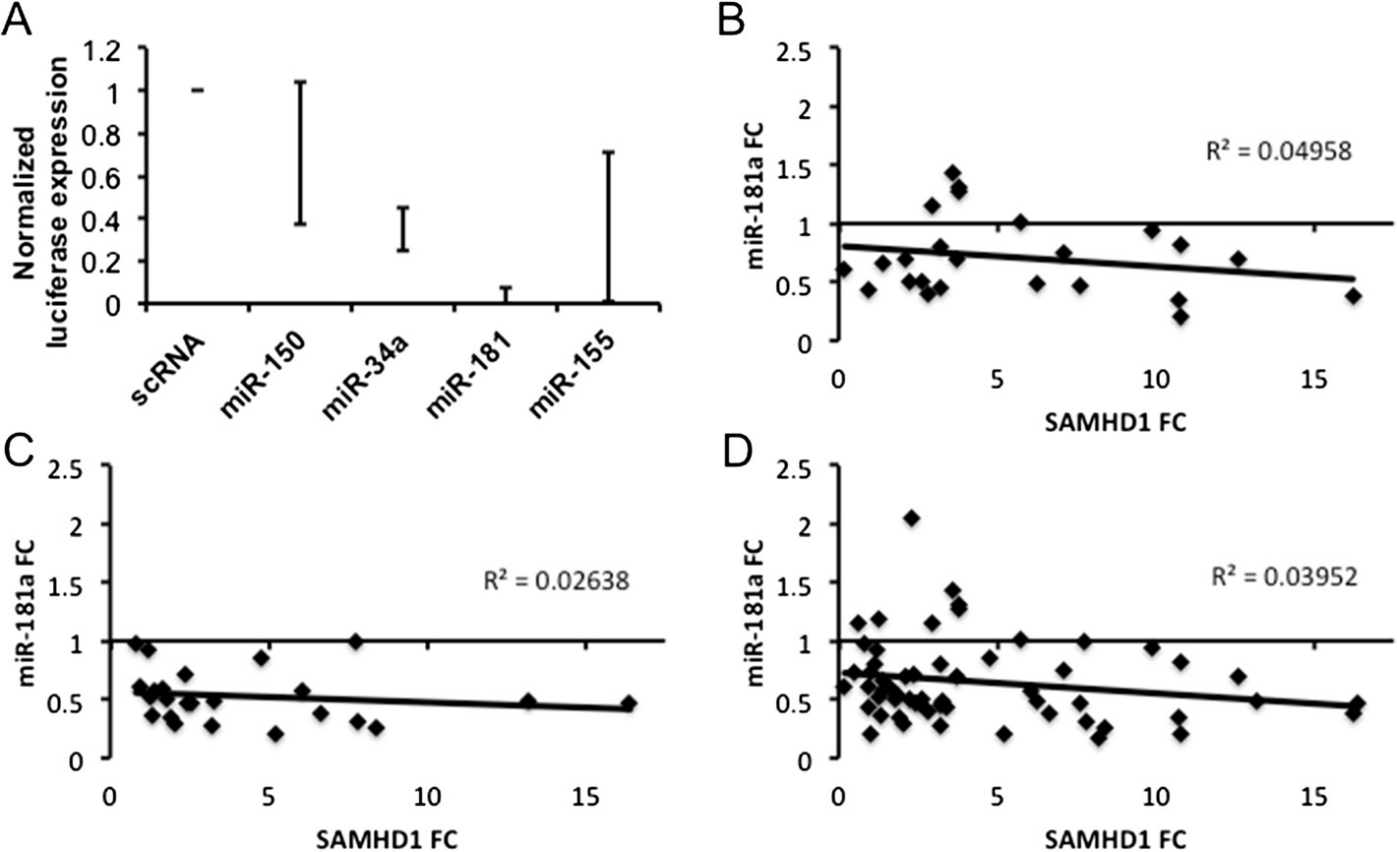
SAMHD1 and miRNAs in vitro and in vivo. **a** miRNAs suppress SAMHD1-linked reporter assay. 293T cells in 96-well plates were transfected with 10 nM scrambled RNA control (scRNA) or 10 nM miRNA mimics (Life Technologies), along with 50 ng luciferase reporter vector containing the human SAMHD1 3′ UTR (SwitchGear Genomics) or empty vector. After 48–72 h, luciferase activity was measured using a Fluoroskan Ascent fluorometer (Thermo Scientific). **b–d** Normalized fold change of thalamic SAMHD1 and miR-181a expression compared with the average of uninfected controls. Total RNA was extracted from the archived thalamus samples from an SIV model of HIV disease. SAMHD1 levels were normalized to the average of GAPDH and actin beta expression using data collected using hydrolysis probe qPCR assays (Life Technologies). Expression of miR-181a (shown) and other miRNAs (including miR-34a and miR-155, not shown) were determined by stem-loop reverse transcription/qPCR assays (Life Technologies) and normalized to U6 snRNA. Correlations of miR-181a and SAMHD1 expression are shown for **b** subjects during acute phase infection (4–14 days post infection), **c** subjects during progression to disease (21–84 days p.i.), and **d** for all samples, including uninfected controls. Each point represents one subject. No correlations were significant (*p* < 0.05) or trended towards significance (*p* < 0.1)

At the same time, we are somewhat uncertain about the extent to which these miRNAs regulate SAMHD1 in vivo. Pilakka-Kanthikeel et al. transfected 50 nM miR-155 and miR-181 mimic or inhibitor and observed effects on SAMHD1 levels in cultured astrocytes [1]. In our hands, 10 nM miR-155 and miR-181—as well as several others including miR-34a and miR-150—reduced the expression of luciferase reporters fused to the SAMHD1 3′ untranslated region (3′ UTR), supporting direct regulation (Fig. 1a). However, the stoichiometries of these “all or nothing” assays are highly artificial, with exposures amounting to millions or tens of millions of oligonucleotides per cell. Additionally, and as reported previously by others [7], we observed significant cell death with miR-181a transfection, a potential explanation for the particularly large reduction in this condition. The authors did not report viability in their populations, but we wonder if this could explain some of the observed effects.

Seeking to determine whether there is a tissue-level, in vivo correlation between SAMHD1 and miRNAs found to regulate the transcript in vitro, we measured SAMHD1 and miRNA expression in a well-characterized simian model of HIV disease [8], including uninfected controls. In the model, thalamic SAMHD1 expression increased significantly after infection (*p* < 0.03), while miR-181a was significantly downregulated (*p* < 0.001). This regulation in opposite directions could be interpreted as consistent with a miR-181a-SAMHD1 interaction. However, further analysis revealed no correlation of miR-181a and SAMHD1 in any of the following groups: acute phase infection (Fig. 1b), progression to disease (Fig. 1c), or all samples, including uninfected controls (Fig. 1d). There was also no correlation of SAMHD1 with levels of miR-155 or miR-34a, which in fact tended to increase during infection (data not shown). These results recall a previous finding in which the RNA binding protein ZFP36 (tristetraprolin) could be suppressed by miRNAs in vitro but showed little evidence of negative correlation with the same miRNAs in vivo [9].

While our findings temper our initial enthusiasm about miRNA-mediated control of SAMHD1, we are not prepared to dismiss it entirely. Although the astrocyte is the most abundant cell type in the brain and appears to support higher levels of SAMHD1 expression than, e.g., macrophages ["Regulation of the SAMHD1 transcript during retroviral infection of the central nervous system," submitted] and microglia [1], the tissue-level profiling we performed may mask changes of miRNA or SAMHD1 expression in specific cells types or even the absence of certain miRNAs in disease-related cells [10]. Similarly, focusing on single miRNA-to-target interactions ignores the regulatory “whole picture,” since a given miRNA-to-target interaction is governed not only by the abundance of each partner but also by the abundance of other partners [11]. Finally, assuming adequate delivery methods, therapeutic antisense manipulation of anti-SAMHD1 miRNAs (for example, to help prevent cell-to-cell transmission during eradication therapy) could be performed at high concentrations not dissimilar to the in vitro manipulations that we and the authors performed. There may will be more to learn about miRNAs and SAMHD1 in the CNS.

## Abbreviations

3′ UTR: 3′ untranslated region
CNS: central nervous system
HIV: human immunodeficiency virus
miRNA: microRNA
SAMHD1: SAM-domain and HD-domain containing protein 1
